# Social Determinants of Health, Depression, and Cardiovascular Health Among Veterans and Civilians: Insights From the 2013–2018 and 2021–2023 National Health and Nutrition Examination Survey

**DOI:** 10.5888/pcd23.260097

**Published:** 2026-09-10

**Authors:** Arum Lim, Chitchanok Benjasirisan, Marian Botchway, Ashwag Alhabodal, Diana-Lyn Baptiste, Jessica Gill, Binu Koirala

**Affiliations:** 1 Johns Hopkins University School of Nursing, Baltimore, Maryland; 2 Medical Nursing Department, Faculty of Nursing, Mahidol University, Bangkok, Thailand; 3 Eck Institute for Global Health, University of Notre Dame, Notre Dame, Indiana; 4 School of Nursing, University of Connecticut, Storrs, Connecticut; 5 Johns Hopkins University School of Medicine, Baltimore, Maryland

## Abstract

**Introduction:**

Emerging evidence supports the relationship among social determinants of health, depression symptoms, and cardiovascular health. Veterans have a disproportionate prevalence of cardiovascular disease and depression, social determinants of health concerns related to socioeconomic status, and challenges with access to healthcare. This study aimed to examine associations of social determinants of health and depression symptoms with cardiovascular health among civilians and veterans and assessed whether these associations differed between the 2 groups.

**Methods:**

Our cross-sectional analysis used 2013–2018 and 2021–2023 National Health and Nutrition Examination Survey data on participants aged 20 years or older without cardiovascular disease. Social determinants of health included demographic characteristics, socioeconomic factors, and access to health care. We used the Patient Health Questionnaire-9 to assess the severity of depression and dichotomized scores as 0 to 5 symptoms (no depression) or 5 or more symptoms (mild to severe depression). We quantified cardiovascular health using Life’s Essential 8 scoring metric (scored from 0 to 100, with higher scores indicating better health status). We applied sample weights in multiple linear regressions.

**Results:**

Among 11,396 participants, 10,470 were civilians (mean age, 46 y; 55% female) and 926 were veterans (mean age, 56 y; 10% female). Mean cardiovascular health score was 71 among civilians and 67 among veterans. Veterans were more likely to have some college, higher financial status, and access to health care. Among both civilians and veterans, poorer cardiovascular health was associated with older age, identifying as non-Hispanic Black or as “other” or multiracial, lower education, lower financial status, and depression symptoms. These associations were more pronounced among veterans who identify as members of racial and ethnic minority populations or have lower financial status.

**Conclusion:**

The magnitude of the links between cardiovascular health and some social determinants of health varied by veteran status, indicating that varied factors may influence health disparities in each group and underscoring the need for tailored strategies to address them.

**Summary**

**What is already known on this topic?**

Adverse social determinants of health, depression symptoms, and veteran status are associated with increased risk of cardiovascular disease.

**What is added by this report?**

Clinical cardiovascular health factors were more strongly associated with age, sex, and race and ethnicity, whereas poorer health behaviors were more strongly associated with education, financial status, and depression symptoms. These associations were more pronounced among veterans who identify as members of racial and ethnic minority populations or have lower financial status.

**What are the implications for public health practice?**

Integrating social determinants of health and depression symptoms into cardiovascular risk assessment may improve identification of civilians and veterans who should be prioritized for cardiovascular disease prevention efforts.

## Introduction

The mind-heart-body connection framework suggested by the American Heart Association recognizes that the mind, heart, and body are intertwined, with social determinants of health (SDoH) affecting a person’s ability to optimize cardiovascular health (CVH) (1,2). SDoH are conditions surrounding peoples’ lives, influencing a range of health outcomes (3). SDoH include socioeconomic status, neighborhood-level racial and ethnic segregation, and built and food environments, all of which are associated with the prevalence of diabetes, hypertension, and elevated body mass index (BMI) (4). Sociodemographic characteristics, including age, sex, race and ethnicity, education, and marital status, are closely intertwined with SDoH and are important indicators of a person’s capacity to achieve and maintain optimal CVH (2).

Veterans have a higher prevalence at younger ages of cardiovascular disease (CVD), including coronary artery disease, heart failure, and stroke, compared with civilians in the same age range (5,6). The increased risk of CVD is caused by traditional risk factors such as higher rates of hypertension, hyperlipidemia, and smoking (6,7); these rates are higher among veterans than among civilians. The prevalence of mental health symptoms such as depression related to combat-related trauma and adverse childhood experiences is also higher among veterans than among civilians (8). One study suggests that veteran status itself is an independent predictor of a 40% higher risk for heart disease, even after adjusting for socioeconomic status, health behaviors, and existing physical and psychological conditions (6). Moreover, health disparities by race and ethnicity, gender, and socioeconomic status are prevalent in veteran populations (9,10). Thus, this study aimed to examine the association of SDoH and depression symptoms with CVH among veterans and civilians and assess how it differs between the 2 groups.

## Methods

### Study design and data source

This study used a cross-sectional design to examine data from the 2013–2018 and 2021–2023 National Health and Nutrition Examination Survey (NHANES) conducted by the National Center for Health Statistics (11). NHANES is a cross-sectional, nationally representative survey of the noninstitutionalized US population of all ages and has been conducted since 1999. It is designed to assess health and nutritional status by computer-assisted personal interviews and physical examination in specially equipped mobile examination centers. NHANES excludes all active duty military personnel and active duty family members but includes people who had served on active duty in the US military (hereinafter, veterans) (11). We downloaded publicly available datasets from the NHANES website and used data on the following: demographic characteristics, health-related data from the interviews (blood pressure and cholesterol, diabetes, health insurance, hospital utilization and access to care, medical conditions, mental health [depression screener], occupation, physical activity, reproductive health, sleep disorders, smoking), dietary interview (total nutrient intakes), health-related data from the examination (blood pressure, body measures), and laboratory data (cholesterol, glycohemoglobin, plasma fasting glucose). The period of the dataset provided the most recent data in the past decade, excluding the survey suspension period caused by the COVID-19 pandemic. The response rates for the examined samples ranged from 25.6% to 68.5% across the survey cycles. This study was exempt from institutional review board review because it involved a secondary analysis of publicly available de-identified data.

### Study population

This study included NHANES participants aged 20 years or older so that we could apply CVH metric scores eligible for adults aged 20 years or older (2). We excluded participants with a self-reported history of CVD (angina, congestive heart failure, coronary heart disease, heart attack, or stroke) because this study focused on CVH before the onset of CVD. Additionally, we excluded women who were pregnant or breastfeeding at the time of the examination, given that some metrics of CVH (eg, cholesterol reference value) are not applicable to this population (12).

### Measurements

#### Cardiovascular health

We used Life’s Essential 8 scoring metric, constructed by the American Heart Association, to measure overall CVH; this metric consists of 4 CVH factors (BMI, blood pressure, blood glucose, and blood lipids) and 4 CVH behaviors (diet, nicotine exposure, sleep, and physical activity) (12). The overall CVH score is an aggregate score ranging from 0 to 100 points, calculated by averaging all 8 component scores (2). A higher score indicates better CVH status. The overall scores were further analyzed separately by CVH factors and CVH behaviors.

#### Veteran status

Veteran status was determined by a self-reported question, “Have you ever served on active duty in the US Armed Forces, Military Reserves, or National Guard?” NHANES respondents who responded yes were considered as having ever served on active duty in the US military.

#### Social determinants of health 

Using the Healthy People 2030 framework (3), we included SDoH from 4 domains: education access and quality (educational level), economic stability (employment and financial status), health care access and quality (health insurance status, having a routine place for health care), and social and community context (age, sex, race and ethnicity, marital status). We did not include the domains of neighborhood and built environment because no appropriate proxy was available in the NHANES dataset. Some variables were defined as dichotomous: sex (male vs female), marital status (married or living with a partner vs unmarried, including never married, widowed, divorced, or separated), employment status (employed vs unemployed), health insurance status (insured vs uninsured), and having a routine place to go for health care (a proxy for health care access) (yes or no). Age in years was used as a continuous variable and further categorized as aged 20 to 49 years, aged 50 to 64 years, and aged 65 years or older. Race and ethnicity were categorized as Mexican American, non-Hispanic Asian, non-Hispanic Black, non-Hispanic White, other Hispanic, or other or multiracial. Educational level was categorized by high school education or less, some college, and college graduate. We used poverty–income ratio (PIR) to determine financial status. To calculate PIR, the midpoint of a person’s family income is divided by the federal poverty threshold for the year. The resulting value was then classified into 3 categories: less than 1, 1 to 1.99, and 2 or more. A PIR less than 1 indicates that the individual’s income falls below the federal poverty level, while a PIR of 1 to 1.99 suggests income between 100% and 199% of the federal poverty level. PIR of 2 or more indicates that the individual’s income is 200% or more of the federal poverty level.

#### Depression symptoms

Depression symptoms were assessed using the Patient Health Questionnaire (PHQ)-9 (13). The PHQ-9 is a screening tool used to assess the severity of depression. Scores ranged from 0 to 27, and predefined cut points were used to assess severity: a score of 0 to 4 indicates no depression, 5 to 9 indicate mild depression, 10 to 14 indicates moderate depression, 15 to 19 indicates moderately severe depression, and a score of 20 to 27 indicates severe depression (13). We dichotomized the PHQ-9 score into having no depression symptoms (PHQ-9 <5) and mild to severe depression symptoms (PHQ-9 ≥5).

### Statistical analysis

We merged NHANES data from 2013–2018 and 2021–2023 and applied sampling weights according to the National Center for Health Statistics guidelines. All analyses were conducted by using R version 4.4.3 (R Foundation). We presented sociodemographic characteristics as mean (SE) for continuous variables and percentages for categorical variables. We then evaluated the mean values of overall CVH score and each component of CVH by military status (veteran vs civilian). Because CVH is significantly correlated with chronological age, we performed age-stratified analyses for overall CVH, CVH factors, CVH behaviors, and each CVH component. We performed survey-weighted *t* tests to compare continuous variables and χ^2^ tests to compare categorical variables. This study used survey-weighted generalized linear models with Gaussian distribution for continuous CVH scores after adjusting covariates for the entire sample and stratified samples by veteran status. Because many SDoH differed significantly between civilians and veterans, we conducted regression analyses separately by veteran status to better characterize the associations between SDoH and CVH in each group. Participants with missing values for any of the SDoH, depression symptoms, or Life’s Essential 8 components were excluded from analysis (Figure). Significance was set as a 2-sided α level of .05. We visually checked linear relationships between continuous variables and used a generalized variance inflation factor to evaluate multicollinearity for categorical variables with more than 2 levels (14). Values of adjusted generalized SE inflation factor greater than 3.16 were deemed to indicate multicollinearity (15). We visually checked independence, normality, and homoscedasticity of residuals after modeling.

**Figure:**
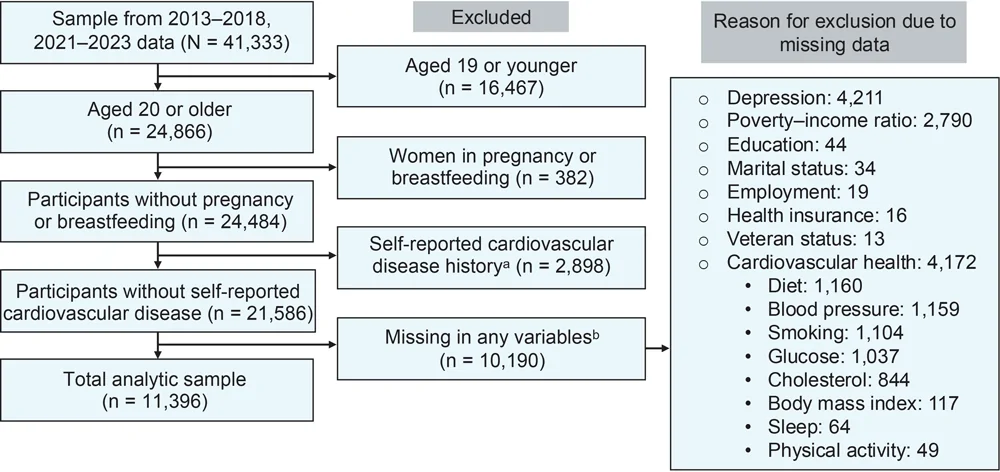
Participant selection flow diagram, analysis of social determinants, depression, and cardiovascular health among veterans and civilians aged 20 years or older, 2013–2018 and 2021–2023 National Health and Nutrition Examination Survey.

## Results

### Sample characteristics

This study included 11,396 participants, of whom 10,470 were civilians and 926 were veterans (Table 1). Civilians were younger than veterans (mean [SE] age, 45.7 [0.3] y vs 55.7 [0.7] y), and women made up a substantially larger proportion of civilians than of veterans (55.0% vs 10.0%). The veteran group had higher proportions of non-Hispanic White and non-Hispanic Black participants, and significantly lower proportions of Mexican American, non-Hispanic Asian, and other Hispanic participants. Compared with civilians, veterans were more likely to have some college, a PIR of 2 or more, health insurance, and a routine place for health care. Severity of depression was similar between the 2 groups.

**Table 1:** Survey-Weighted Sociodemographic Characteristics of Participants Aged ≥20 Years Without Cardiovascular Disease, 2013–2018 and 2021–2023 National Health and Nutrition Examination Survey

Characteristic	Total (N = 11,396)	Military status		
		Civilian	Veteran^a^	*P* value^b^
Unweighted sample size (%)	**11,396 (100.0)**	**10,470 (91.9)**	**926 (8.1)**	**—**
**Age, mean (SE), y**	46.5 (0.3)	45.7 (0.3)	55.7 (0.7)	<.001
**Age group, %**				
20–49 y	56.5	58.3	35.7	<.001
50–64 y	27.6	27.7	26.3	
≥65 y	15.9	13.9	38.0	
**Sex, %**				
Male	48.7	45.0	90.0	<.001
Female	51.3	55.0	10.0	
**Race and ethnicity, %**				
Mexican American	8.3	8.7	3.5	<.001
Non-Hispanic Asian	4.9	5.3	1.0	
Non-Hispanic Black	9.8	9.7	11.7	
Non-Hispanic White	66.8	66.0	76.2	
Other Hispanic	6.3	6.6	3.7	
Other and multiracial	3.8	3.8	3.9	
**Marital status, %**				
Married or living with partner	64.3	63.7	71.4	<.001
Unmarried^c^	35.7	36.3	28.6	
**Educational level, %**				
High school education or less	35.0	35.3	31.2	<.001
Some college	32.2	31.4	41.7	
College graduate	32.8	33.3	27.1	
**Employment status, %**				
Employed	67.0	68.1	54.4	<.001
Unemployed	33.0	31.9	45.6	
**Poverty**–**income ratio^d^ %**				
≥2	68.8	68.4	73.2	<.001
1–1.99	18.6	18.6	18.9	
<1	12.6	13.0	7.9	
**Health insurance, %**				
Insured	86.2	85.7	91.9	<.001
Uninsured	13.8	14.3	8.1	
**Have a routine place for health care, % **				
Yes	82.2	81.7	87.3	.002
No	17.8	18.3	12.7	
**Depression symptoms^e^**				
Mean scores (SE) of symptoms	3.0 (0.06)	3.1 (0.06)	2.8 (0.20)	.16
No depression (0–4 points)	77.0	77.1	76.7	.85
Mild to severe depression (5–27 points)	23.0	22.9	23.3	

Abbreviation: —, not applicable.

a People who had served on active duty in the US military.

b Determined by survey-weighted *t* tests and χ^2^ tests. Significance was set as a 2-sided α = .05.

c Includes never married, widowed, divorced, and separated.

d Less than 1 = below the federal poverty level; 1–1.99 = 100%–199% above the federal poverty level; ≥2 = 200% or more above federal poverty level.

e Assessed using Patient Health Questionnaire-9 (13).

### Unadjusted CVH scores by age group and veteran status

Among participants aged 20 to 49 years, the mean overall CVH score was significantly lower among veterans than among civilians (71.4 vs 74.9) (Table 2). Veterans had lower BMI, blood pressure, blood lipid, and nicotine exposure scores but higher physical activity scores. Among participants aged 50 to 64 years, veterans had a lower mean overall CVH score (65.2 vs 68.5), worse BMI and nicotine exposure scores, but better physical activity scores. Among participants aged 65 or older, overall CVH in the civilian and veteran groups did not differ, although veterans had better blood pressure and lipid scores and poorer diet and nicotine exposure scores.

**Table 2:** Survey-Weighted Cardiovascular Health Scores,^a^ by Age Group and Military Status, Among Civilians and Veterans^b^ Aged ≥20 Years Without Cardiovascular Disease (N = 11,396), 2013–2018 and 2021–2023 National Health and Nutrition Examination Survey

Age group	Total (N = 11,396)	Military status		
		Civilian (n = 10,470)	Veteran (n = 926)	*P* value^c^
**Aged 20–49 y**				
No. of survey participants	5,954	5,690	264	—
Overall CVH	74.7 (0.32) ​	74.9 (0.33)	71.4 (0.92)	<.001
CVH factors	77.1 (0.38)	77.4 (0.40)	71.0 (1.31)	<.001
Body mass index	60.4 (0.88)	60.8 (0.92)	53.5 (2.15)	.003
Blood pressure	82.1 (0.51)	82.4 (0.52)	75.3 (2.40)	.005
Blood glucose	93.1 (0.34)	93.2 (0.36)	91.7 (1.16)	.23
Blood lipids	72.7 (0.64)	73.2 (0.65)	62.7 (2.18)	<.001
CVH behaviors	72.3 (0.40)	72.3 (0.41)	72.0 (1.02)	.73
Diet	53.3 (0.45)	53.4 (0.48)	50.8 (1.46)	.11
Nicotine exposure	72.3 (0.96)	72.6 (0.99)	66.1 (2.88)	.03
Sleep	85.2 (0.44)	85.3 (0.46)	83.4 (1.49)	.23
Physical activity	78.5 (0.74)	78.0 (0.73)	87.6 (2.35)	<.001
**Aged 50–64 y**				
No. of survey participants	3,168	2,901	267	—
Overall CVH	68.2 (0.45)	68.5 (0.46)	65.2 (1.00)	.002
CVH factors	65.9 (0.55)	66.2 (0.55)	62.5 (1.36)	.007
Body mass index	56.8 (1.03)	57.4 (1.03)	50.2 (2.91)	.02
Blood pressure	65.3 (0.87)	65.6 (0.87)	61.4 (2.65)	.11
Blood glucose	80.9 (0.72)	81.1 (0.73)	78.0 (2.22)	.16
Blood lipids	60.6 (0.91)	60.7 (0.91)	60.2 (3.04)	.88
CVH behaviors	70.5 (0.61)	70.7 (0.63)	68.0 (1.43)	.06
Diet	53.3 (0.65)	53.6 (0.63)	49.4 (2.58)	.10
Nicotine exposure	74.8 (1.25)	75.5 (1.32)	66.4 (3.14)	.009
Sleep	84.7 (0.58)	85.1 (0.65)	80.2 (2.23)	.05
Physical activity	69.3 (1.29)	68.8 (1.32)	76.1 (2.98)	.01
**Aged ≥65 y**				
No. of survey participants	2,274	1,879	395	—
Overall CVH	68.0 (0.41)	68.0 (0.45)	67.9 (0.90)	.92
CVH factors	64.0 (0.78)	63.4 (0.55)	66.7 (1.03)	.006
Body mass index	60.8 (0.84)	60.8 (0.97)	61.0 (1.72)	.91
Blood pressure	53.8 (1.00)	52.5 (1.12)	59.3 (2.22)	.009
Blood glucose	76.0 (0.79)	76.0 (0.84)	75.7 (1.54)	.84
Blood lipids	65.5 (0.91)	64.2 (1.00)	70.8(1.81)	.002
CVH behaviors	71.9 (0.55)	72.6 (0.57)	69.1 (1.24)	.009
Diet	53.2 (0.69)	54.4 (0.83)	47.9 (1.77)	.003
Nicotine exposure	81.9 (0.78)	83.8 (0.85)	73.7 (1.87)	<.001
Sleep	87.7 (0.65)	87.7 (0.73)	87.8 (1.43)	.92
Physical activity	65.0 (2.07)	64.6 (1.49)	66.9 (3.43)	.52

Abbreviations: —, does not apply; CVH, cardiovascular health.

a CVH was quantified using the American Heart Association’s Life’s Essential 8 metric, ranging from 0 to 100. Values are presented as score (SE), and a higher score indicates better health status.

b People who had served on active duty in the US military.

c Determined by survey-weighted *t* tests. Significance was set as a 2-sided α = .05.

### SDoH related to overall CVH, CVH factors, and CVH behaviors

We observed lower overall CVH scores among participants aged 50 years or older (vs those aged 20–49 y) and among non-Hispanic Black participants and other and multiracial participants (vs non-Hispanic White participants) (Table 3). Compared with college-educated participants, those with a high school education or less had 7.19 points lower overall CVH (95% CI, −8.11 to −6.27), and those with a PIR less than 1 had 1.96 points lower overall CVH (95% CI, −3.07 to −0.84) than those with a PIR of 2 or more. Female sex and non-Hispanic Asian race and ethnicity were associated with higher overall CVH scores. Scores for CVH factors declined with age, whereas the scores for CVH behaviors did not. We observed lower scores for CVH factors among Mexican American, non-Hispanic Black, other Hispanic, and other and multiracial participants (vs non-Hispanic White) and among those with less than a college degree (vs some college or high school graduate or less), while poorer scores for CVH behaviors were associated with being non-Hispanic Black, being unmarried, having lower educational attainment, being unemployed, having a PIR less than 2, and lacking health insurance. Depression symptoms were associated with lower overall CVH score, with stronger associations for CVH behaviors than for CVH factors.

**Table 3:** Survey-Weighted Multiple Linear Regressions for Cardiovascular Health^a^ in the Overall Sample, Participants Aged ≥20 Years Without Cardiovascular Disease (N = 11,396), 2013–2018 and 2021–2023 National Health and Nutrition Examination Survey^b^

Variable	Overall CVH, β (95% CI)	CVH factors, β (95% CI)	CVH behaviors, β (95% CI)
Intercept	79.32 (78.52 to 80.11)	78.46 (77.21 to 79.71)	80.17 (79.16 to 81.17)
**Age, y**			
20–49	[Reference]	[Reference]	[Reference]
50–64	−6.45 (−7.36 to −5.54)	−10.95 (−12.30 to −9.60)	−1.95 (−3.06 to −0.84)
≥65	−6.72 (−7.62 to −5.82)	−13.15 (−14.44 to −11.87)	−0.29 (−1.59 to 1.01)
**Sex**			
Male	[Reference]	[Reference]	[Reference]
Female	2.26 (1.60 to 2.93)	3.74 (2.80 to 4.69)	0.79 (−0.04 to 1.61)
**Race and ethnicity**			
Mexican American	−0.90 (−1.69 to −0.11)	−5.15 (−6.46 to −3.83)	3.34 (2.22 to 4.46)
Other Hispanic	0.20 (−1.03 to 1.43)	−2.07 (−3.53 to −0.61)	2.47 0.63 to 4.31)
Non-Hispanic Asian	2.06 (0.68 to 3.44)	2.32 (0.30 to 4.35)	1.79 (0.58 to 3.01)
Non-Hispanic Black	−3.41 (−4.10 to −2.71)	−4.86 (−6.02 to −3.70)	−1.95 (−2.89 to −1.02)
Non-Hispanic White	Reference	Reference	[Reference]
Other and multiracial	−2.59 (−4.61 to −0.56)	−3.09 (−5.91 to −0.27)	−2.08 (−4.20 to 0.04)
**Marital status**			
Married or living with partner	[Reference]	[Reference]	[Reference]
Unmarried^c^	0.45 (−0.26 to 1.15)	1.91 (0.90 to 2.92)	−1.02 (−1.76 to −0.28)
**Educational level**			
College graduate	[Reference]	[Reference]	[Reference]
Some college	−5.24 (−6.20 to −4.28)	−4.46 (−5.67 to −3.25)	−6.01 (−7.24 to −4.78)
High school education or less	−7.19 (−8.11 to −6.27)	−5.07 (−6.31 to −3.84)	−9.31 (−10.32 to −8.30)
**Employment status**			
Employed	[Reference]	[Reference]	[Reference]
Unemployed	−1.00 (−1.72 to −0.27)	−0.15 (−1.05 to 0.75)	−1.84 (−2.89 to −0.80)
**Poverty**–**income ratio^d^**			
≥2	[Reference]	[Reference]	[Reference]
1–1.99	−1.23 (−2.12 to −0.35)	−0.20 (−1.51 to 1.11)	−2.26 (−3.45 to −1.08)
<1	−1.96 (−3.07 to −0.84)	0.45 (−1.24 to 2.13)	−4.36 (−5.71 to −3.01)
**Health insurance**			
Insured	[Reference]	[Reference]	[Reference]
Uninsured	−0.64 (−1.63 to 0.35)	1.35 (−0.11 to 2.81)	−2.63 (−3.76 to −1.51)
**Have a routine place for health care**			
Yes	[Reference]	[Reference]	[Reference]
No	1.48 (0.56 to 2.40)	3.18 (1.92 to 4.44)	−0.22 (−1.46 to 1.02)
**Depression symptoms^e^**			
No depression (0–4 symptoms)	[Reference]	[Reference]	[Reference]
Mild to severe depression (5–27 symptoms)	−3.82 (−4.55 to −3.09)	−2.70 (−3.86 to −1.55)	−4.94 (−5.72 to −4.15)

Abbreviations: β, adjusted estimated coefficient (adjusted for all variables listed in the table); CVH, cardiovascular health.

a CVH quantified using the American Heart Association’s Life’s Essential 8 metric, ranging from 0 to 100; a higher score indicates better health status.

b Survey-weighted generalized linear models with Gaussian distribution were used.

c Includes never married, widowed, divorced, and separated.

d Less than 1 = below the federal poverty level; 1–1.99 = between 100%–199% above the federal poverty level; ≥2 = 200% or more above the federal poverty level.

e Assessed using the Patient Health Questionnaire-9 (13).

### SDoH related to overall CVH stratified by veteran status

Associations observed in the overall sample were largely preserved among civilians. Among veterans, older age (aged 50–64 y and ≥65 y), identifying as non-Hispanic Black or other and multiracial, lower educational attainment, and having a PIR less than 1 were associated with poorer overall CVH (Table 4). Veterans with a PIR less than 1 had significantly lower CVH scores (β = −4.75, 95% CI, −7.03 to −2.47) than those with a PIR of 2 or more; a similar pattern was observed among civilians (β = −1.82, 95% CI, −2.93 to −0.71). Direct comparisons across models were limited given the stratified analytic approach.

**Table 4:** Survey-Weighted Multiple Linear Regressions for Overall Cardiovascular Health^a^ Among Participants Aged ≥20 Years Without Cardiovascular Disease, Stratified by Veteran Status, 2013–2018 and 2021–2023 National Health and Nutrition Examination Survey^b^

Variable	Civilians, β (95% CI) (n = 10,470)	Veterans, β (95% CI) (n = 926)
Intercept	**79.49 (78.62 to 80.35)**	**76.66 (74.09 to 79.23)**
**Age, y**		
20–49	[Reference]	[Reference]
50–64	−6.50 (−7.43 to −5.57)	−4.97 (−7.90 to −2.04)
≥65	−7.02 (−8.00 to −6.04)	−3.36 (−6.36 to −0.37)
**Sex**		
Male	[Reference]	[Reference]
Female	2.15 (1.42 to 2.88)	3.36 (0.10 to 6.61)
**Race and ethnicity**		
Mexican American	−0.89 (−1.75 to −0.02)	−1.09 (−4.46 to 2.28)
Non-Hispanic Asian	2.05 (0.63 to 3.47)	0 (−5.44 to 5.44)
Non-Hispanic Black	−3.28 (−4.05 to −2.51)	−4.25 (−6.45 to −2.05)
Non-Hispanic White	Reference	Reference
Other Hispanic	0.30 (−0.96 to 1.57)	−1.69 (−5.34 to 1.96)
Other and multiracial	−2.41 (−4.64 to −0.18)	−5.26 (−8.43 to −2.09)
**Marital status**		
Married or living with partner	[Reference]	[Reference]
Unmarried^c^	0.40 (−0.36 to 1.16)	0.72 (−1.29 to 2.72)
**Education level**		
College graduate	[Reference]	[Reference]
Some college	−5.14 (−6.19 to −4.10)	−5.22 (−7.70 to −2.74)
High school education or less	−7.39 (−8.36 to −6.42)	−4.55 (−7.28 to −1.82)
**Employment status**		
Employed	[Reference]	[Reference]
Unemployed	−0.92 ( −1.66 to −0.19)	−1.80 (−3.85 to 0.25)
**Poverty**–**income ratio^d^**		
≥2	[Reference]	[Reference]
1–1.99	−1.32 (−2.23 to −0.41)	0.20 (−2.34 to 2.74)
<1	−1.82 (−2.93 to −0.71)	−4.75 (−7.03 to −2.47)
**Health insurance**		
Insured	[Reference]	[Reference]
Uninsured	−0.63 (−1.65 to 0.39)	−1.47 (−4.98 to 2.05)
**Have a routine place for health care**		
Yes	Reference	Reference
No	1.46 (0.56 to 2.35)	2.22 (−1.13 to 5.57)
**Depression symptoms^e^**		
No depression (0–4 symptoms)	[Reference]	[Reference]
Mild to severe depression (5–27 symptoms)	−3.77 (−4.55 to −2.99)	−4.10 (−6.96 to −1.24)

Abbreviation: β, adjusted estimated coefficient (adjusted for all variables listed in the table).

a Cardiovascular health quantified using the American Heart Association’s Life’s Essential 8 metric, ranging from 0 to 100; a higher score indicates better health status.

b Survey-weighted generalized linear models with Gaussian family were used.

c Includes never married, widowed, divorced, separated.

d Less than 1 = below the federal poverty level; 1–1.99 = 100%–199% above the federal poverty level; ≥2 = 200% or more above the federal poverty level.

e Assessed using the Patient Health Questionnaire-9 (13).

## Discussion

Our study found that in the total sample, certain SDoH were associated with poorer overall CVH, particularly among participants identifying as members of racial and ethnic minority groups, participants with lower educational attainment, and participants with lower financial status. After stratifying overall CVH into CVH factors and CVH behaviors in the total sample, racial and ethnic minority status was more strongly associated with lower CVH factor scores, whereas lower educational attainment, lower financial status, and depression symptoms were associated with lower CVH behavior scores. The overall trend remained after stratification by veteran status. The associations of SDoH with poorer overall CVH were especially pronounced among veterans identifying as other and multiracial and among veterans with a PIR less than 1.

Our findings indicated that veterans with a PIR less than 1 have poorer CVH than those with a PIR of 2 or greater, and the magnitude of the gap in CVH was greater than in civilians. Because the US Department of Veterans Affairs provides several programs to reduce adverse SDoH for veterans, such as GI Bill benefits for education, home loans, and health-related services (10), veterans are less likely than nonveterans in general to live in poverty and they have better access to health care (16). However, despite these provisions, living in poverty was significantly associated with poor CVH among veterans, suggesting that these efforts may be insufficient to address CVH disparities among veterans most vulnerable to socioeconomic disadvantage. Literature also reports heterogeneity in CVH factors and behaviors across various racial and ethnic groups, including African Americans, Latinos, and Asians (17–19), as well as different associations between SDoH and CVH by race and ethnicity (20). However, existing evidence is often not applicable to those identifying as other or multiracial because members of racial and ethnic minority populations were excluded or aggregated into bigger groups. Our study showed that the CVH scores among veterans identifying as other or multiracial were approximately 5 points lower overall than scores among non-Hispanic White veterans. This finding may indicate greater health disparities in CVH among veterans who are members of racial and ethnic minority groups after adjusting for other SDoH, and it highlights the necessity of disaggregated data and targeted structural interventions, particularly given that the other and multiracial groups represent a heterogeneous population whose CVH disparities may stem from distinct social and structural exposures. Veterans from racial and ethnic minority populations are more likely to have adverse SDoH, such as living in poverty, housing instability, and food insecurity, compared with non-Hispanic White veterans. Our study did not examine housing instability and food insecurity, but these factors may have confounded the association of identifying as a member of a racial or ethnic minority population with CVH. SDoH interact across multiple factors, collectively exerting cumulative downstream effects on CVH through diverse social, physiological, and behavioral pathways (20). Thus, it is important to consider the intersectionality among SDoH, including mental health, to examine the associations with CVH.

Examining CVH among veterans showed different patterns for each CVH component according to age group. Compared with civilians, veterans had better physical activity in the youngest and middle-aged groups but worse nicotine exposure in all age groups. In the youngest group, veterans had poorer blood pressure and lipid scores than civilians had, but in the oldest group, veterans had better scores for both. This finding was aligned with previous evidence that veterans had a higher risk for CVD at younger ages (aged 25–65 y) but a lower risk at older ages (aged ≥66 y) than nonveterans (5). A possible explanation is a survival bias, given that veterans had a higher mortality rate than civilians in the 55 to 64 age group, and it decreased to a similar level as civilians in veterans aged 65 years or older. Moreover, selection bias may have occurred if we excluded a greater proportion of veterans aged 65 years or older with CVD than the same age group of civilians with CVD. Another possible explanation is the influence of SDoH on CVH factors. Because our results were unadjusted, more favorable social determinants, such as higher socioeconomic status and greater educational attainment, may have acted as protective factors, contributing to better CVH factor scores among veterans aged 65 years or older. Previous studies reported that young veterans who had recently left active duty were more likely than older veterans to have lower grades in high school, less educated parents, and worse socioeconomic status when they enlisted (7,21). Furthermore, veterans in the youngest group had more recent experiences in military service and may therefore have been likely to have more acute stress responses affecting blood pressure and blood lipids. A study found that older veterans (aged ≥50 y) were less likely to have elevated depression and anxiety symptoms than nonveterans in the same age group (22), which may indicate that the effect of traumatic exposure may be attenuated over time or that older veterans have developed coping strategies, such as resilience or posttraumatic growth, that positively affected long-term health conditions (23). Moreover, combat experiences, their severity and length, and their impact on psychological symptoms vary by war (22,24). Recent wars in Iraq and Afghanistan have been reported to be more psychologically stressful than older wars (24), which may lead to a more detrimental effect on blood pressure and blood lipids in the youngest veterans through greater autonomic response and chronic inflammation (25).

By examining CVH rather than CVD as the outcome, our study aimed to clarify how adverse SDoH and depression symptoms contribute to future CVD risk and to identify which factors and behaviors most strongly drive declines in CVH scores. This approach provides actionable insights to inform targeted CVD prevention strategies. We found that poorer CVH behavior scores were associated with depression symptoms, lower educational attainment, and lower PIR, whereas CVH factors were more strongly associated with age, sex, and race and ethnicity, factors that may influence CVH through biological aging processes and genetic susceptibility. Because CVH factors, including hypertension, diabetes, hyperlipidemia, and obesity, often increase with age and are influenced by genetic susceptibility (26,27), people with these conditions typically require medical management, including pharmacotherapy and clinical monitoring, especially as they age. In contrast, CVH behaviors are more amenable to modification through education, social and financial support, and engagement with health care professionals, including interventions addressing psychological factors that influence health behaviors. Designing and implementing interventions that integrate clinical risk factor management by health care professionals with behavior modification and self-management is essential for preventing CVD. Such approaches may be particularly beneficial for individuals with limited access to health care, including those living in rural areas or those living in poverty, by addressing the intersectional effects of SDoH.

Having depression symptoms was significantly associated with overall CVH among both veterans and civilians after adjusting for SDoH. Previous studies suggested that the relationship between depression and higher CVD prevalence may be mediated through depression-related behaviors, including reduced physical activity and poorer sleep quality (28,29). Our stratified analyses of CVH factors and behaviors showed lower CVH behavior scores among individuals with depression symptoms. Although the cross-sectional design of our study limits causal inference, a prospective cohort study suggests that depression may contribute to worsening CVH behaviors over time (28), and a link between depression and increased risk for CVD has been demonstrated (30). However, residual confounding from unmeasured psychological conditions such as posttraumatic stress disorder and substance use disorders may play a role. Thus, longitudinal studies that include additional confounders are warranted to clarify the relationships between depression and CVH and compare CVD outcomes between civilians and veterans.

### Limitations and strengths

Our study has several limitations. First, the small sample size after stratification may have limited statistical power to detect significant associations among veterans. Despite the reduction in sample size, analyses were stratified by veteran status rather than modeled using interaction terms or adjustment alone, because veteran status is associated with multiple SDoH rather than a single determinant, and stratification allowed clear examination of group-specific relationships. Second, the NHANES dataset lacks detailed information on veterans’ service experiences, such as the number of deployments, length of service, and military rank, making it difficult to account for heterogeneity within the veteran group. Nevertheless, this dataset provides comparable data on civilians and postservice veterans, allowing meaningful group comparisons. We did not account for other psychological and physical conditions, such as posttraumatic stress disorder and traumatic brain injury, which often co-occur with depression and are more prevalent among veterans than civilians. Trauma-related disorders and substance use may also disproportionately affect CVH among veterans (10,31,32). Considering these factors in future studies could improve the understanding of CVH determinants in this population. Lastly, the gap spanning the COVID-19 pandemic between the 2017–2018 and 2021–2023 survey cycles may have introduced heterogeneity in SDoH between and within the 2 groups.

Despite these limitations, our study has strengths. First, it used a nationally representative dataset with a weighted survey approach in both veterans and civilians. Second, by stratifying groups based on veteran status, we identified differences in CVH patterns between veterans and civilians by age group and identified poorer CVH factors and behaviors among veterans from racial and ethnic minority groups and those living in poverty. Third, we examined the associations of SDoH with CVH factors and behaviors separately. These findings can ultimately inform the underlying pathways of CVD onset and develop a tailored approach to preventing CVD in individuals with adverse SDoH. Our findings indicate that additional research should evaluate the detailed context of military services in larger veteran populations, addressing trauma-related psychological and physical disorders, and focusing on each SDoH and their intersectionality to examine any detrimental or protective effects on CVH.

### Conclusion

Our study consolidated the association between SDoH and CVH and showed that CVH disparities among racial and ethnic minority groups and poor financial status existed in veteran populations. Our findings emphasize the need for risk screening and stratification, including SDoH assessment, to identify individuals who should be prioritized for effective risk management and intervention to prevent CVD. Recommendations include developing tailored approaches for CVD risk management that consider multiple factors, including demographic characteristics, education, and depression symptoms, in addition to veteran status.
